# Lupus and NMOSD: The Blending of Humoral Autoimmunity

**DOI:** 10.1155/2020/8820071

**Published:** 2020-10-15

**Authors:** Maria Goretti S. Ochi, Samantha C. Shapiro, Esther Melamed

**Affiliations:** ^1^Department of Medicine, Dell Medical School at the University of Texas at Austin, Austin, TX, USA; ^2^Department of Medicine, Division of Rheumatology, Dell Medical School at the University of Texas at Austin, Austin, TX, USA; ^3^Department of Neurology, Dell Medical School at the University of Texas at Austin, Austin, TX, USA

## Abstract

Systemic lupus erythematous (SLE) is a chronic autoimmune disease that can target any organ of the body. It may coexist with other autoimmune neurologic conditions such as neuromyelitis optica spectrum disorder (NMOSD). NMOSD, previously known as Devic's disease, is an autoimmune inflammatory disorder of the central nervous system (CNS) that targets the spinal cord, optic nerves, and certain brain regions. Most current evidence suggests that NMOSD is best described as a CNS astrocytopathy. While these diseases share several immunosuppressive treatment options, timely diagnosis of NMOSD is critical as patients may benefit from treatment tailored specifically to NMOSD as opposed to SLE. Steroids, plasmapheresis, intravenous immunoglobulin, cyclophosphamide, azathioprine, mycophenolate mofetil, and rituximab are used to treat both SLE and NMOSD. However, there are several new therapies (inebilizumab, eculizumab, and satralizumab) recently approved specifically for use in NMOSD. In this case series, we report on three patients with coexisting SLE and NMOSD. We describe a 31-year-old woman who suffered an NMOSD flare after 11 years of clinical remission in the context of receiving an influenza vaccination; her SLE remained quiescent on hydroxychloroquine. Next, we describe a 52-year-old woman with emergence of neurologically devastating seropositive NMOSD in the setting of active treatment for SLE with intravenous cyclophosphamide, oral steroids, and hydroxychloroquine. Last, we describe a 48-year-old woman with emergence of seronegative NMOSD in the setting of SLE that was well-controlled on azathioprine and hydroxychloroquine. These cases illustrate the importance of accurate diagnosis and targeted treatment of NMOSD when coexisting with SLE.

## 1. Introduction

SLE is a chronic autoimmune disease with multiorgan involvement. The etiology of SLE is likely multifactorial with genetic, hormonal, immunologic, and environmental factors contributing. The reported prevalence of SLE is 20 to 150 cases per 100,000; in women, rates vary from 164 (Caucasian) to 406 (African American) per 100,000 [1–3]. Neurologic and psychiatric symptoms occur in 10 to 80 percent of patients during the course of illness [4–8]. Transverse myelitis (TM) is one of the most common neurologic manifestations of SLE and is characterized by longitudinal inflammation of the spinal cord with motor or sensory loss and/or loss of rectal and urinary bladder sphincter control [9]. While symptoms of TM may be the initial feature of SLE [10], the onset may coincide with other manifestations of active lupus [11].

Over the last few years, it has become apparent that neurologic symptoms of SLE may coexist with those of NMOSD. NMOSD, previously known as Devic's disease, is a CNS astrocytopathy with damage mediated by the humoral immune system [12–15]. The most common clinical features are optic neuritis, longitudinally extensive transverse myelitis (LETM), area postrema syndrome, and other brainstem and cerebral events. Historically, NMOSD was considered an opticospinal variant of multiple sclerosis [16]. However, it is clear that NMOSD is in fact a separate disease with both clinical heterogeneity and heterogeneity in immunopathology. The identification of anti-aquaporin-4 antibodies (AQP4-IgG) and myelin oligodendrocyte glycoprotein antibodies (MOG-IgG) has furthered increased our understanding in this regard [12, 17]. Approximately 20 to 30 percent of patients with clinical characteristics of NMOSD are AQP4-lgG negative, but 30 percent of those seronegative for AQP4-IgG have detectable MOG-IgG [16, 18]. The seronegative NMOSD subset of patients tend to be younger, less frequently female, more responsive to steroids, and less likely to relapse [16].

Proper identification of NMOSD as a comorbid diagnosis with SLE is critical, as the coexistence of both disorders may alter therapeutic management. All SLE patients should be treated with hydroxychloroquine (barring no contraindications) as it has been shown to reduce lupus flares [19]. When SLE is active despite chronic hydroxychloroquine therapy, many other immunosuppressive agents may be added depending on organ involvement. These include but are not limited to steroids, azathioprine, mycophenolate mofetil, cyclophosphamide, and rituximab [19]. In NMOSD, acute attacks are treated with high-dose intravenous methylprednisolone, with addition of plasma exchange (PLEX) in cases of a poor response. Preventive immunosuppressive therapy options in NMOSD include azathioprine, rituximab, and mycophenolate mofetil [20], as well as more recently FDA approved therapies: eculizumab (monoclonal antibody against complement C5), satralizumab (monoclonal antibody against the IL-6 receptor), and inebilizumab (monoclonal antibody against CD19-positive B-cells) [21, 22, 23].

As patients may have coexistent SLE and NMOSD, it is critical to consider both diseases when selecting therapy in order to ensure best clinical outcomes. In the following case series, we describe three patients with coexisting SLE and NMOSD and discuss the clinical management in each case.

## 2. Case Presentations

### 2.1. Case 1

**Figure 1:**
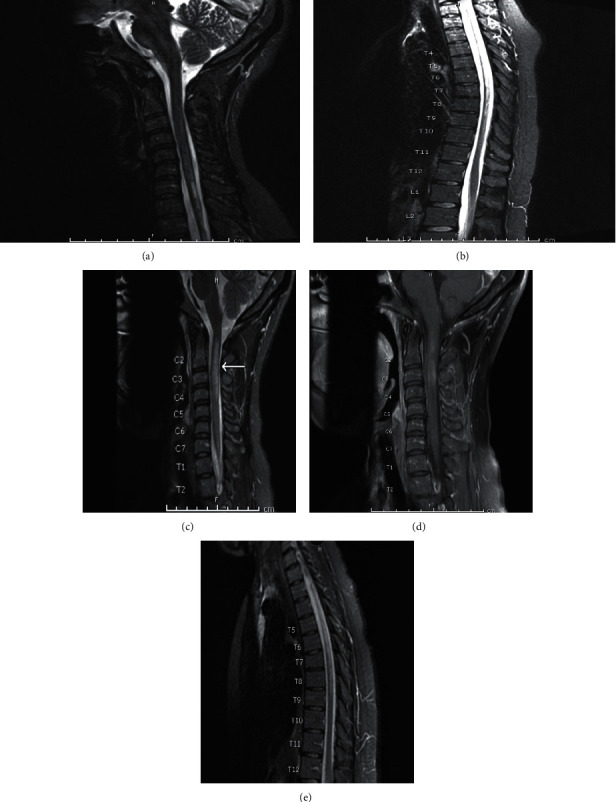
T2WI of cervical and thoracic spine of a 31-year-old African American woman with NMOSD and SLE. In 2018, cervical (a) and thoracic spine (b) demonstrated a C6 hyperintense lesion extending to T8 (a, b), which was unchanged from a prior spine MRI of the patient (not shown). In 2019, there was a new C2–C4 longitudinal lesion noted on the C spine on T2WI (c) with associated patchy postcontrast enhancement on T1WI (d) and an ongoing hyperintense lesion in the T spine extending to T8 (e).

A 31-year-old African American woman with NMOSD and SLE in remission presented with progressive paresthesia and right-sided weakness one month after receiving an influenza vaccination. In 1999, she was diagnosed with multiple sclerosis (MS) on the basis of CNS imaging and cerebrospinal fluid studies. During a period of sixteen years, she was treated with high-dose steroids and interferon-beta. Her diagnosis was later revised to NMOSD when she tested positive for AQP4-IgG in 2007. Her disease was in remission by 2011, with residual neurologic deficits of no light perception in the left eye. MRI of the brain and total spine in 2015 and 2018 (Figure 1) showed stable C6-T8 myelomalacia with no active inflammation. In 2017, she was diagnosed with SLE on the basis of positive anti-nuclear, anti-double stranded deoxyribonucleic acid, anti-Smith, and anti-ribonucleoprotein antibodies, leukopenia, hypocomplementemia, alopecia, and inflammatory arthritis. She was started on hydroxychloroquine for improvement in inflammatory arthritis, white blood cell counts, and complement levels. SLE was in remission by 2018.

She had received yearly influenza vaccinations since 2015 without issue. However, hours after receiving her vaccine in 2019, she developed numbness and paresthesia in the posterior neck and right upper back. Paresthesia worsened over the following three weeks with spread to the right arm, right hand, and right leg. By the time she was evaluated in rheumatology clinic (four weeks after vaccination), her sensory symptoms had spread to include her left leg. Physical exam was notable for decreased sensation in the right V3 distribution of the face, right bicep weakness graded at 4/5, right extensor hallucis longus weakness graded at 3/5, and right foot drop. She also had 3+ asymmetric hyperreflexia in the right bicep and right patellar tendon. She was referred to the emergency room for further evaluation. Upon arrival, MRI with contrast of the brain, cervical, and thoracic spine revealed an acute enhancing lesion extending from C1 to C4 (Figure 1). She was admitted for management of an NMOSD relapse. She received methylprednisolone 1000 mg IV daily for a total of five doses, plasmapheresis daily for 5 exchanges, and one dose of rituximab 1000 mg IV following plasmapheresis with complete return of neurologic exam to baseline. She had no serologic or clinical evidence of a contemporaneous SLE flare, and thus, a single dose of rituximab for NMOSD treatment, as opposed to the 2 doses commonly used in SLE treatment, was administered. Hydroxychloroquine was continued without changes. Rituximab 1000 mg IV every 6 months was continued as maintenance therapy for NMOSD.

### 2.2. Case 2

**Figure 2:**
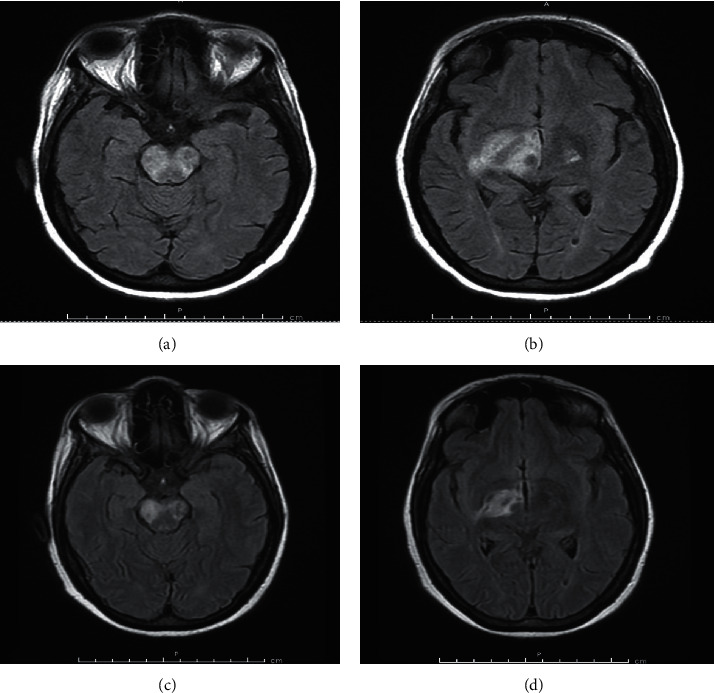
FLAIR brain MRI of a 52-year-old African American woman with active SLE demonstrating edematous lesion in the brainstem (a) extending to the basal ganglia (b) at initial hospital presentation in July 2019. Edema and lesion size decreased posttreatment with methylprednisolone, PLEX, and cyclophosphamide (c) and (d) FLAIR fluid-attenuated inversion recovery.

A 52-year-old African American woman with active SLE presented with altered mental status and rapidly progressive focal neurologic deficits. She was diagnosed with SLE in 2015 on the basis of positive anti-nuclear and anti-double stranded deoxyribonucleic acid antibodies, leukopenia, hypocomplementemia, alopecia, oral ulcers, and inflammatory arthritis. She was started on hydroxychloroquine but was minimally compliant with therapy. In November 2018, she developed nephrotic range proteinuria. Renal biopsy confirmed lupus nephritis (ISN/RPS class III). Prednisone and mycophenolate mofetil were added to her regimen, but noncompliance persisted due to multiple social issues. In May 2019, she presented to rheumatology clinic with fatigue, dysarthria, headache, and worsening inflammatory arthritis. She was admitted to the hospital for further evaluation. She was diagnosed with lupus cerebritis on the basis of cerebrospinal fluid pleocytosis (WBC 28 cells/uL) with elevated protein (46 mg/dL), as well as diffuse T2 hyperintensities involving the thalamus, midbrain, pons, and medulla. CSF flow cytometry was normal. She clinically improved on pulse dose methylprednisolone and was discharged on oral steroids and monthly intravenous cyclophosphamide. CSF and serum AQP4-IgG were drawn during admission and resulted as positive after discharge. She was compliant with medications and did well until July 2019, when she was found down and unresponsive at home. At this time, MRI brain demonstrated increased thalamocapsular and pontomesencephalic edema (Figure 2). Neurologic exam was notable for complete facial diplegia, anarthria, mixed ophthalmoparesis with right gaze preference, dense paraplegia of the left upper and lower extremities graded at 0/5, and neck flexor and extensor weakness graded at 0/5. Percutaneous gastrostomy tube placement was required due to aspiration risk. Neuroimmunology was consulted during this hospitalization, and her diagnosis was revised to concomitant SLE and NMOSD given positive CSF and serum AQP4-IgG. She was treated with pulse dose methylprednisolone and PLEX for 5 days, followed by a fourth cycle of IV cyclophosphamide. Repeat MRI brain showed only slight improvement in previously noted lesions (Figure 2). MRI C/T spine remained normal without evidence of myelitis. She was discharged to a skilled nursing facility with a prolonged steroid taper. Hydroxychloroquine was continued, and cyclophosphamide was stopped in favor of rituximab 1000 mg IV on day 0 and day 14 every 6 months in order to prevent NMOSD relapse, as well as SLE flare. By March 2020, the patient had significant improvement in her neurologic exam with only mild residual dysarthria although dense left hemiplegia was unchanged. Percutaneous gastrostomy tube was removed, and she was able to transition to a pureed diet.

### 2.3. Case 3

**Figure 3:**
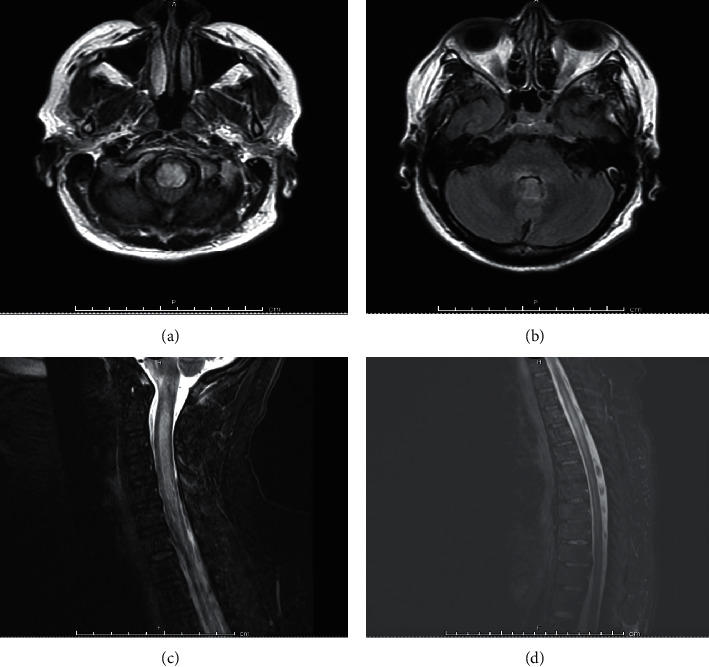
FLAIR brain MRI of a 48-year-old Hispanic woman with SLE with noted medullary lesion extending to the periaqueductal gray matter (a, b) and with diffuse lesions in the C and T spine (c, d).

A 48-year-old Hispanic woman with well-controlled SLE presented with headache and diplopia. SLE was diagnosed in 2016 on the basis of positive anti-nuclear, anti-double stranded deoxyribonucleic acid, anti-Smith, anti-ribonucleoprotein, anti-Ro, and anti-La antibodies, hypocomplementemia, alopecia, inflammatory arthritis, hemolytic anemia, leukopenia, and oral ulcers. She was admitted to the hospital in September 2019 for fever, headache, diplopia, and diffuse body pain. CSF studies demonstrated low glucose (33 mg/dL), high protein (189 mg/dL), and pleocytosis (WBC 508 cells/uL) with neutrophilic predominance concerning for bacterial meningitis. MRI of the brain was normal. She was treated with antibiotics for presumed bacterial meningitis with normalization of CSF studies on repeat testing four days later. CSF and blood cultures were negative. She was continued on hydroxychloroquine, azathioprine, and low-dose prednisone without changes. She was readmitted in November 2019 for intractable nausea and vomiting, fever, and severe headache. CSF studies again demonstrated low glucose (34 mg/dL), high protein (148 mg/dL), and pleocytosis (WBC 294 cells/uL) with neutrophilic predominance concerning for recurrent bacterial meningitis—similar to her previous admission. She was treated with broad-spectrum antibiotics without improvement. CSF cultures remained negative. Headache, nausea, and vomiting persisted, and she developed new diplopia. Repeat MRI brain demonstrated new abnormal lower brainstem and periaqueductal gray signal abnormality; in retrospect, her clinical presentation was area postrema syndrome due to NMOSD (Figure 3). Ophthalmologic exam was normal, and there were no optic nerve abnormalities on MRI orbit. AQP4-IgG and MOG-IgG were negative. Neuroimmunology was consulted, and the patient was diagnosed with seronegative NMOSD with coexistent SLE. She received PLEX for 5 days and pulse dose methylprednisolone. Rituximab 1000 mg IV on day 0 and day 14 was administered for long-term treatment of SLE and prevention of future NMOSD relapses. Hydroxychloroquine was continued, and azathioprine was stopped. She was discharged on a prolonged glucocorticoid taper. By April 2020, both SLE and NMOSD remained well-controlled without relapse.

## 3. Discussion

Here, we present three cases of SLE and NMOSD that highlights the importance of timely diagnosis of these coexistent conditions in order to guide therapeutic management. SLE is an autoimmune disease mediated by autoantibodies and immune complex deposition [24]. For example, lupus nephritis is caused by immune complex deposition and subsequent complement activation in the kidney, leading to tissue damage. Anti-nuclear antibodies (ANA) are the hallmark of disease and may interact with nuclear antigens expressed on cell surfaces in various organs, triggering cell injury and death through complement activation and/or cell penetration [21]. Though rare, patients with SLE can develop brain and brainstem inflammation and myelitis. SLE-mediated myelitis may present similarly to NMOSD, with MRI demonstrating longitudinal spinal involvement with cord swelling and hyperintense lesions [25].

In NMOSD, complement deposition and demyelination may involve multiple spinal cord segments, the brain, and the optic nerves with associated axonal loss, perivascular lymphocytic infiltration, and vascular proliferation [12]. One of the target antigens in NMOSD is AQP4, a water channel protein highly concentrated in spinal cord gray matter, periaqueductal and periventricular regions, and astrocytic foot processes at the blood-brain barrier [26, 27]. Another known antibody in NMOSD is MOG-IgG. MOG is a glycoprotein localized on the surface of the myelin sheath, cell body, and processes of oligodendrocytes [20]. MOG-IgG is found in about 30 percent of patients who are seronegative for AQP4-IgG [16]. NMOSD is frequently associated with other systemic autoimmune disorders, particularly those that are humorally mediated such as SLE, Sjogren's syndrome, hypothyroidism, and myasthenia gravis [28–30]. Seventy percent of patients with NMOSD make at least one other autoantibody in addition to AQP4-IgG, alluding to the frequency of comorbid autoimmune diagnoses [25].

A recent study investigated the frequency of autoantibodies classically associated with neuropsychiatric SLE (NPSLE), like ANA and anti-dsDNA, in patients with NMOSD [31]. Eighty-eight percent of patients with coexisting NMOSD had AQP4-IgG in the serum, whereas AQP4-IgG was only present in the serum of three percent of patients with NPSLE alone. In addition, no AQP4-IgG's were found in SLE patients without neuropsychiatric symptoms [31]. MOG-IgG antibodies were only detected in patients with NMOSD who were negative for AQP4-IgG. The authors concluded that patients with demyelinating NPSLE should be tested for AQP4-IgG and MOG-IgG in order to help identify comorbid SLE and NMOSD. AQP4-IgG can be considered diagnostic for NMOSD, while none of the other antibodies were diagnostic of demyelinating NPSLE [31].

Some NMOSD treatments are similar to those used in SLE although there are important differences. High-dose intravenous steroids are first-line therapy for acute exacerbations of both NMOSD and SLE. However, the choice of steroid-sparing agent should be tailored to the active underlying disease. SLE is treated with hydroxychloroquine in addition to other immunosuppressive agents depending on the involved organ system [20]. In NMOSD, recent data suggest that rituximab, eculizumab, satralizumab, and inebilizumab are most effective in preventing relapse although these therapies have been mainly studied in AQP4-IgG seropositive patients [20, 21, 22, 23].

## 4. Conclusion

We describe three cases of coexistent SLE and NMOSD, with several differences in clinical presentation. In the first case, the patient experienced an NMOSD flare in the setting of quiescent SLE and a known NMOSD diagnosis. In the second case, the patient had an initial NMOSD flare in the setting of active SLE. In the third case, the patient had an initial NMOSD flare in the setting of well-controlled SLE. These cases illustrate that SLE and NMOSD may coexist, and this may not be uncommon. The clinical manifestations of myelitis are similar in both SLE and NMOSD, and MRI findings of longitudinal cord swelling and hyperintensity in central cord regions may be seen with both disorders. It is critical to determine which disease is clinically active in order to appropriately tailor therapy. Though both disorders share similar neurologic manifestations, antibody testing for AQP4-IgG and MOG-IgG may help identify coexisting NMOSD. Based on our experience caring for these three patients and studies to date, we recommend that clinicians consider testing for AQP4-IgG and MOG-IgG in SLE patients with neurologic signs and symptoms. In seronegative cases where NMOSD is suspected but not supported by antibody testing, we recommend that these patients still be treated for NMOSD if imaging and CSF findings meet diagnostic criteria for this entity. It is imperative to identify coexisting SLE and NMOSD as treatment differs, and inappropriate treatment can lead to irreversible and severe neurologic outcomes.

## Data Availability

Not applicable.
